# *SMARCA4* mutations and expression in lung adenocarcinoma: prognostic significance and impact on the immunotherapy response

**DOI:** 10.1002/2211-5463.13899

**Published:** 2024-09-25

**Authors:** Yuming Zhang, Dantong Sun, Weizhong Han, Zhen Yang, Yongzhi Lu, Xuchen Zhang, Yongjie Wang, Chuantao Zhang, Ning Liu, Helei Hou

**Affiliations:** ^1^ Precision Medicine Center of Oncology The Affiliated Hospital of Qingdao University, Qingdao University China; ^2^ Department of Medical Oncology National Cancer Center/National Clinical Research Center for Cancer/Cancer Hospital, Chinese Academy of Medical Sciences and Peking Union Medical College Beijing China; ^3^ Department of Respiratory Medicine The Affiliated Hospital of Qingdao University China; ^4^ Department of Pathology The Affiliated Hospital of Qingdao University, Qingdao University China; ^5^ Department of Oncology Qingdao Municipal Hospital China; ^6^ Department of Thoracic Surgery The Affiliation Hospital of Qingdao University China; ^7^ Department of Oncology The Affiliated Hospital of Qingdao University China

**Keywords:** immunotherapy sensitivity, lung adenocarcinoma, prognosis, *SMARCA4*, SWI/SNF complex

## Abstract

The switch/sucrose non‐fermenting (SWI/SNF) complex family includes important chromatin‐remodeling factors that are frequently mutated in lung adenocarcinoma (LUAD). However, the role of one family member, *SMARCA4*, in LUAD prognosis and immunotherapy sensitivity remains unclear. In the present study, 6745 LUAD samples from the cBioPortal database were used to analyze the relationships between *SMARCA4* mutations and patient prognoses and clinical characteristics. Additionally, we examined the correlation between *SMARCA4* mutations and prognosis in patients treated with immunotherapy using two immune‐related datasets. *SMARCA4* mutations and low expression were associated with shorter survival, and mutations were associated with a high tumor mutational burden and high microsatellite instability. *SMARCA4* mutations were accompanied by *KRAS*, *KEAP1*, *TP53* and *STK11* mutations. No significant difference was observed in the immunotherapy response between patients with and without *SMARCA4* mutations. When *KRAS* or *STK11* mutations were present, immunotherapy effectiveness was poorer; however, when both *SMARCA4* and *TP53* mutations were present, immunotherapy was more effective. Furthermore, low *SMARCA4* expression predicted a higher immunophenoscore, and *SMARCA4* expression was correlated with certain immune microenvironment features. Taken together, our results suggest that *SMARCA4* mutations and low expression might be associated with poor LUAD prognosis. Additionally, immunotherapy efficacy in patients with *SMARCA4* mutations depended on the co‐mutant genes. Thus, *SMARCA4* could be an important factor to be considered for LUAD diagnosis and treatment.

## Graphical abstract

*SMARCA4* has a high mutation frequency in lung adenocarcinoma (LUAD) and its mutation might lead to low gene expression. Our study suggests that *SMARCA4* mutations and low expression might be associated with poor LUAD prognosis. Furthermore, *SMARCA4* mutations and expression level might influence the efficacy of immunotherapy in LUAD patients. *SMARCA4* could serve as a potential prognostic biomarker for LUAD.

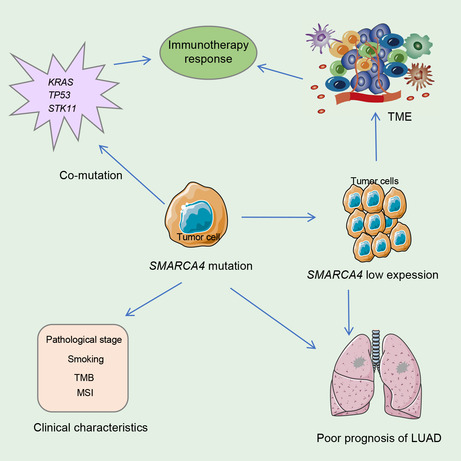

**Abbreviations**
ESTIMATE: Estimation of STromal and Immune cells in MAlignant Tumors using Expression data
GO: Gene Ontology
ICIs: immune checkpoint inhibitors
KEGG: Kyoto Encyclopedia of Genes and Genomes
K–M: Kaplan–Meier
LUAD: lung adenocarcinoma
MSI: microsatellite instability
NSCLC: non‐small cell lung cancer
OS: overall survival
PD‐1: programmed cell death 1
PD‐L1: programmed cell death ligand 1
PFS: progression‐free survival
PPI: protein–protein interaction
SWI/SNF: switch/sucrose non‐fermenting
TCGA: The Cancer Genome Atlas
TMB: tumor mutational burden
TME: tumor microenvironment

Lung cancer is one of the most common malignant tumors worldwide. According to GLOBOCAN 2020 research data, it has the second highest incidence globally and is the leading cause of cancer‐related death [1]. Lung adenocarcinoma (LUAD) is the predominant histological type and accounts for approximately 40% of all lung cancer cases [2].

Immunotherapy mainly includes immune checkpoint inhibitors (ICIs), oncolytic virus therapy, cytokine therapies, adoptive cell therapy and vaccines, among others [3, 4]. ICIs, as one of the most important immunotherapy approaches, have become standard treatments for advanced non‐small cell lung cancer (NSCLC) and have achieved significant therapeutic effects. Here, the most widely targeted molecules are programmed cell death 1/programmed cell death ligand 1 (PD‐1/PD‐L1) and cytotoxic T‐lymphocyte‐associated antigen 4 [5, 6, 7]. Many novel immunotherapy drugs have been approved for clinical use by the US Food and Drug Administration. However, a subset of patients still does not benefit from immunotherapy [8, 9]. Therefore, the exploration of new biomarkers to predict the prognosis of patients with LUAD is urgent.

Switch/sucrose non‐fermenting (SWI/SNF) complexes are important chromatin‐remodeling factors that play crucial roles in cellular processes, such as development, proliferation, differentiation and DNA repair. Moreover, they reshape the nucleosome structure in an ATPase‐dependent manner and thus exert significant effects on biological processes [10, 11, 12]. Mammalian SWI/SNF complexes are composed of 12–15 subunits, with ATPase being the core subunit [13]. However, in malignant tumors, the SWI/SNF family member mutation frequency is estimated to be approximately 20%, and these mutations disrupt complex functions [14]. Among SWI/SNF family members, *ARID1A* and *SMARCA4* genes have the highest mutation rates, and most of these mutations are inactivating, leading to decreased protein expression. Currently, most studies indicate that *ARID1A* is a tumor suppressor gene, and mutations and downregulation of the expression of this gene are associated with a poor prognosis for patients with tumors [15, 16, 17]. Moreover, *ARID1A* mutations or low expression can improve the tumor microenvironment (TME) and enhance the response of the tumor to immunotherapy [18, 19, 20]. However, previous findings regarding SMARC family molecules, especially *SMARCA4*, are controversial. Specifically, some studies suggest that *SMARCA4* functions as a tumor suppressor and that mutations and low expression in this gene are associated with a poor prognosis for patients with tumors [21, 22, 23, 24], whereas the opposite conclusions have been reached in other studies [25, 26, 27]. Similarly, differing viewpoints on the relationship between *SMARCA4* mutations or expression and the sensitivity of tumors to immunotherapy have been published [28, 29, 30, 31, 32]. In the present study, we focused on LUAD and explored the effect of SWI/SNF subunit family members on the prognosis of patients with LUAD. We specifically explored the molecular characteristics related to *SMARCA4* mutations in LUAD and assessed the impact of *SMARCA4* mutations or expression on patient survival and immunotherapy effectiveness.

## Materials and methods

### Study patients

We selected 14 LUAD datasets from the cBioPortal database (https://www.cbioportal.org), which included 6745 LUAD samples. Basic clinical information of the patients, including their age, sex and smoking history, was collected. Additionally, we selected two lung cancer immunotherapy datasets from the cBioPortal database, containing data from 487 patients, including 366 LUAD patients who received immunotherapy, as reported in previous studies [33, 34]. Response Evaluation Criteria in Solid Tumors (RECIST) version 1.1[35] was used to assess the efficacy of ICI therapy. Progression‐free survival (PFS) was evaluated as the duration during which patients maintained a response to treatment (manifested as a complete response, a partial response, or stable disease). The POPLAR and OAK studies compared the therapeutic efficacy of the PD‐L1 inhibitor atezolizumab and docetaxel in patients with NSCLC, and thus data from these studies were included in our analysis [36, 37]. The clinicopathological features of the patients and treatment efficacy were evaluated as previously described [38]. The POPLAR and OAK studies included a total of 1137 patients, among whom 86 patients (7.6%) harbored *SMARCA4* mutations, with six patients having more than one mutation. The Cancer Genome Atlas (TCGA) database (https://portal.gdc.cancer.gov) was utilized to analyze the correlation between gene expression and immune cell infiltration or the immune response in LUAD.

### Survival and statistical analyses

Survival analysis was conducted using the Kaplan–Meier (K–M) plotter method, and log‐rank tests were employed to detect significant differences. In total, 1379 samples with relatively complete clinical data in the cBioPortal database were subjected to screening for the multivariate analysis. Currently, no clear threshold has been defined to distinguish high and low tumor mutational burdens (TMBs). In this study, TMB‐high was defined as ≥ 10 muts·Mb^−1^ and TMB‐low was defined as < 10 muts·Mb^−1^, which is consistent with the criteria of several randomized lung cancer trials and was also applied in the study of a US Food and Drug Administration‐approved anti‐PD‐1 therapy for solid tumors of unknown histological types [39, 40, 41]. The relationship between *SMARCA4* expression and LUAD patient survival was analyzed using the K–M plotter online tool (https://kmplot.com). The clinical characteristics of patients in the mutation and wild‐type groups were compared via Student's *t*, chi‐squared or Fisher's exact tests. All results were plotted using r, version 4.2.1 (R Foundation, Vienna, Austria), photoshop (Adobe Inc., Mountain View, CA, USA) or prism, version 8.0 (GraphPad Software Inc., San Diego, CA, USA).

### Enrichment analysis and protein–protein interaction network of *SMARCA4*‐related genes

We screened the top 200 genes associated with *SMARCA4* in the cBioPortal datasets and performed gene set enrichment analysis using the online tool Sangerbox (http://www.sangerbox.com/index.html). The c5.go.bp.v7.4.symbols.gmt and c2.cp.kegg.v7.4.symbols.gmt subsets were used as the background sets. The enrichment analysis was conducted using the r package ‘clusterProfiler’ [42]. Additionally, the STRING online tool (https://string‐db.org) was used to construct a protein–protein interaction (PPI) network.

### Immune cell infiltration and gene expression analysis of the TCGA‐LUAD cohort

The r package ‘CIBERSORT’ [43] was used to assess the fraction of 22 tumor‐infiltrating immune cells in the TCGA‐LUAD cohort. Using support vector regression and deconvolution algorithms, CIBERSORT can describe and accurately calculate the proportions of different immune cell components in bulk tumor samples. The samples were divided into two groups according to the median gene expression value, and the proportions of infiltrating immune cells in the two groups were compared. We used the Estimation of STromal and Immune cells in MAlignant Tumors using Expression data (ESTIMATE) algorithm to obtain the immune score, stromal score, and the ESTIMATE score of each sample [44]. We also downloaded the TCGA‐LUAD immune score file from the TCIA (https://tcia.at/home) to analyze the relationship between the *SMARCA4* expression level and immunotherapy sensitivity.

## Results

### SWI/SNF‐family genes are mutant at a high frequency in LUAD and these gene mutations are associated with a poor prognosis

**Fig. 1:**
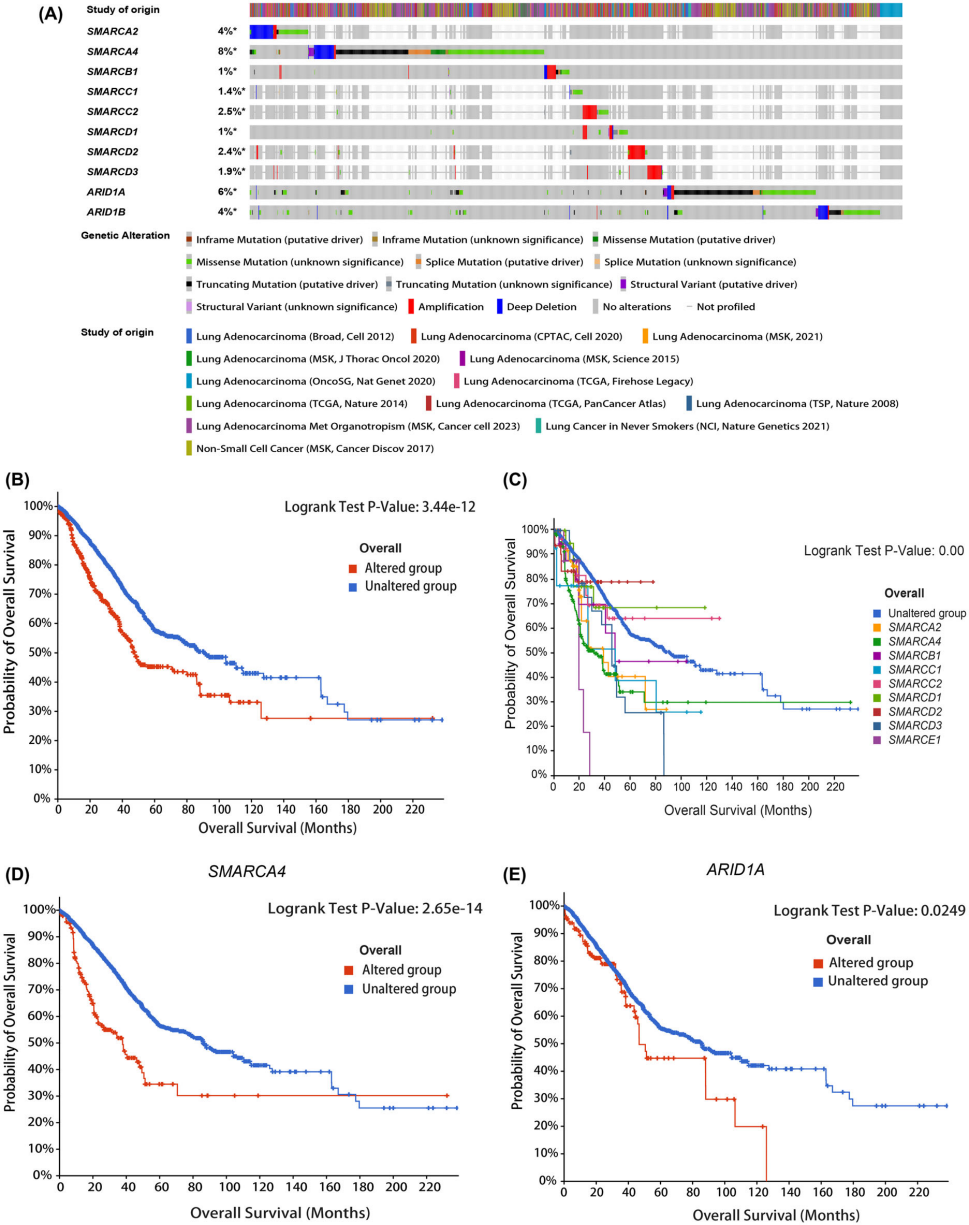
Mutations in SWI/SNF family genes are strongly associated with the prognosis of patients with lung adenocarcinoma (LUAD). (A) Frequency of SWI/SNF family member mutations in patients with LUAD based on the cBioPortal database. *: Not all samples are profiled. (B) Association between global mutations in 10 members of the SWI/SNF family and the overall survival (OS) of patients with LUAD. (C) Analysis of the associations between individual gene mutations and OS in patients with LUAD. (D) Relationship between *SMARCA4* mutations and the prognosis of patients with LUAD. (E) Relationship between *ARID1A* mutations and the prognosis of patients with LUAD.

Through an analysis of the cBioPortal dataset, the mutation frequency of SWI/SNF‐family genes in LUAD was found to be approximately 20% (1358/6745 samples). The mutation rate of *SMARCA4* was the highest at 8%, followed by that of *ARID1A* (6%), *SMARCA2* (4%) and *ARID1B* (4%) (Fig. 1A). We subsequently investigated the relationships between gene mutations and overall survival (OS) in LUAD patients. Patients with genomic mutations had a worse OS than those without mutations (mOS: 47.77 months *vs*. 90.90 months) (Fig. 1B). Additionally, survival analysis showed that only mutations in *SMARCA4*, *SMARCA2* and *ARID1A* were significantly associated with OS (Fig. 1C–E, Fig. S1). In the studied cohort, patients with *SMARCA4* mutations had the poorest prognosis, with a median OS of 25.54 months.

### *SMARCA4* mutations result in decreased expression and truncating mutations account for a large proportion of driver mutations

**Fig. 2:**
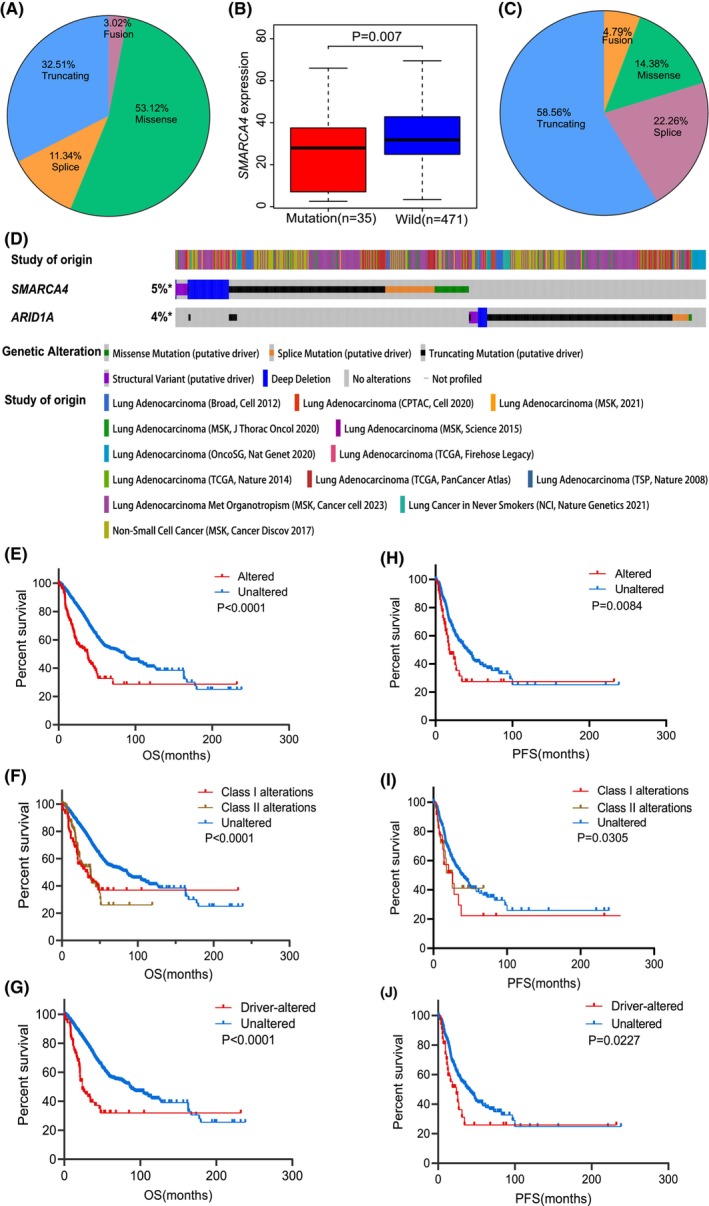
Relationships between *SMARCA4* mutation status and its gene expression level, as well as the prognosis of patients with lung adenocarcinoma (LUAD). (A) Proportion of *SMARCA4* mutation types in LUAD. (B) mRNA expression of *SMARCA4* in the gene‐mutant and wild‐type groups (minimum to maximum, Wilcoxon rank‐sum test, mutation: *n* = 35, wild: *n* = 471). (C) Proportion of LUAD patients with different *SMARCA4*‐driver mutation types. (D) Distributions of different *SMARCA4* and *ARID1A* driver mutations. *: Not all samples are profiled. (E–G) Relationships between *SMARCA4* mutations and overall survival (OS) in patients with LUAD. (H–J) Relationships between *SMARCA4* mutations and progression‐free survival (PFS) in patients with LUAD.

Next, we investigated the clinical significance of *SMARCA4* mutations in LUAD. Among the 6745 LUAD samples, 529 had *SMARCA4* mutations, including missense mutations (53.12%), truncating mutations (frameshift, in‐frame or nonsense mutations) (32.51%), splice site mutations (11.34%) and gene fusions (3.02%) (Fig. 2A). By analyzing *SMARCA4* expression in TCGA database, *SMARCA4* mRNA expression levels were decreased in the group with mutations (Fig. 2B). After excluding non‐driver mutations, the proportion of mutation types changed, and the results were as follows: truncating mutations (58.56%), splice site mutations (22.26%), missense mutations (14.38%) and fusions (4.79%) (Fig. 2C). Notably, among the driver mutations, *SMARCA4* mutations and *ARID1A* mutations were mutually exclusive (Fig. 2D).

*SMARCA4* mutations can be divided into two categories: (a) class 1 alterations, including truncating mutations, fusions and homozygous deletions, which result in protein loss and gene inactivation, and (b) class 2 alterations, including all missense mutations and other variants of unknown significance [45, 46]. In the present study, we analyzed the relationship between *SMARCA4* mutations and patient prognosis. The OS of the *SMARCA4*‐altered group was poorer than that of the unaltered group (mOS: 37.7 months *vs*. 85.3 months) (Fig. 2E), and the same trend was observed when mutations were grouped into class 1 (mOS = 34.29 months) and class 2 (mOS = 37.8 months) (Fig. 2F). The same conclusion was reached when non‐driver mutations were excluded (Fig. 2G). Similarly, the *SMARCA4*‐ altered group had a shorter PFS than the unaltered group (17.62 months *vs*. 41.26 months) (Fig. 2H–J).

### *SMARCA4* mutations are associated with smoking and an advanced pathological stage and are accompanied by *TP53*, *KRAS*, *KEAP1* and *STK11* mutations but are mutually exclusive with EGFR mutations

**Fig. 3:**
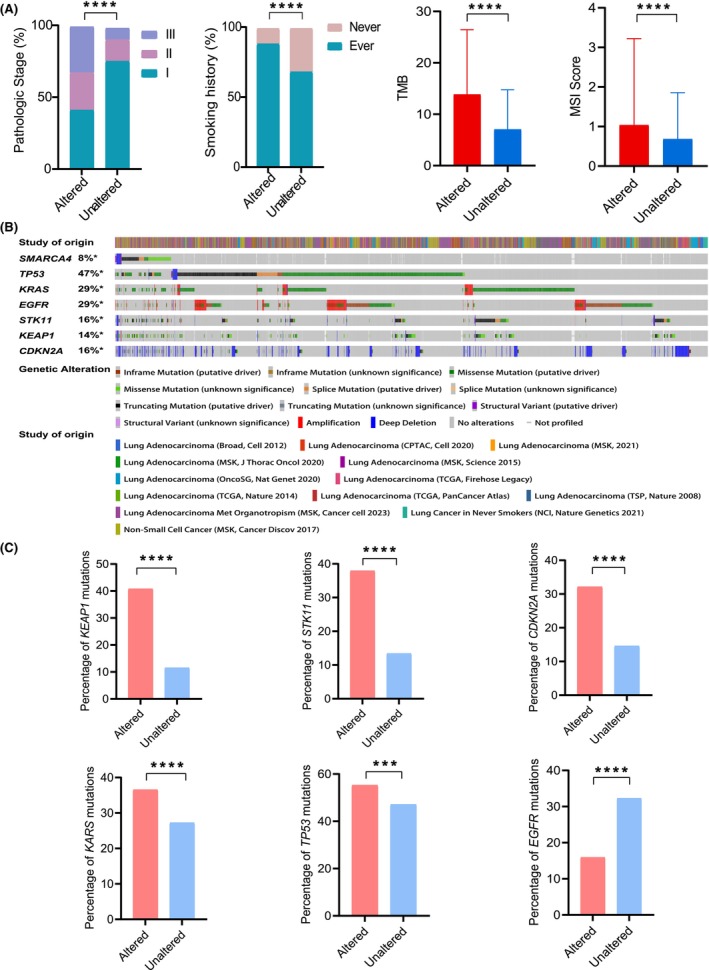
Correlation between *SMARCA4* mutations and clinical characteristics. (A) Association between *SMARCA4* mutations and the clinical features of lung adenocarcinoma (LUAD). The statistical method used for pathologic stage (altered: *n* = 34, unaltered: *n* = 570) and smoking history (altered: *n* = 143, unaltered: *n* = 1512) was the chi‐squared test. The statistical methods used for TMB (mean ± SD, altered: *n* = 515, unaltered: *n* = 5871) and MSI (mean ± SD, altered: *n* = 232, unaltered: *n* = 2277) was Student's *t*‐test. (B) Gene mutation profiles highly associated with *SMARCA4* mutations in LUAD. *: Not all samples are profiled. (C) Association between *SMARCA4* mutations with *KEAP1* (*SMARCA4* altered: *n* = 216; *SMARCA4* unaltered: *n* = 688), *STK11* (*SMARCA4* altered: *n* = 201; *SMARCA4* unaltered: *n* = 800), *CDKN2A* (*SMARCA4* altered: *n* = 167; *SMARCA4* unaltered: *n* = 871), *KRAS* (*SMARCA4* altered: *n* = 190; *SMARCA4* unaltered: *n* = 1628), *TP53* (*SMARCA4* altered: *n* = 294; *SMARCA4* unaltered: *n* = 2815), and *EGFR* (*SMARCA4* altered: *n* = 84; *SMARCA4* unaltered: *n* = 1929) mutations in LUAD (chi‐squared test). ***P < 0.001; ****P < 0.0001.

To further investigate the biological significance of *SMARCA4* mutations in LUAD, we analyzed the relationships between the *SMARCA4* mutation status and clinical characteristics. The results showed a direct correlation between *SMARCA4* mutations and an advanced pathological stage, patient smoking history, TMB and microsatellite instability (MSI) score (*P* < 0.0001) (Fig. 3A). Approximately 32.35% of the *SMARCA4*‐altered samples were from stage III disease, whereas only 8.95% of the *SMARCA4*‐unaltered samples were from stage III disease, and 75.96% were from stage I disease. A significantly greater percentage of patients in the *SMARCA4*‐altered group (88.11%) had a history of smoking compared with that in the *SMARCA4*‐unaltered group (68.58%). Furthermore, the *SMARCA4*‐altered group presented with an increased TMB and MSI scores.

Through an analysis of genomic mutations, mutations in *TP53* (55.26%), *KEAP1* (40.75%), *STK11* (37.92%) and *KRAS* (36.54%) exhibited the highest co‐occurrence rates with *SMARCA4* mutations among the commonly mutant genes in lung cancer. In the *SMARCA4*‐unaltered group, the mutation rate of *EGFR* was highest (32.27%). However, *KEAP1*, *STK11* and *CDKN2A* were more commonly mutant in the *SMARCA4*‐altered group (*P* < 0.001) (Fig. 3B,C).

### *SMARCA4* mutations are an independent prognostic factor for LUAD

We further screened and collated samples with relatively comprehensive clinical data from the cBioPortal cohort (1379 patients) for univariate and multivariate analyses. Baseline patient data are shown in Table 1. Correlation analysis showed that *SMARCA4* mutations were significantly correlated with sex, stage, TMB and smoking history. In addition, a univariate analysis revealed that the *SMARCA4* mutation status, stage, age, sex, smoking history and TMB were prognostic factors for LUAD. Furthermore, a multivariate analysis revealed that the *SMARCA4* mutation status, stage, age and smoking history were independent prognostic factors for LUAD (Table 2).

**Table 1:** Clinical baseline data from the cBioPortal cohort.

Clinical parameters	Total (*N*)		*SMARCA4*		*P*‐value
			Mutant (*N* = 81) (%)	Wild‐type (*N* = 1298) (%)	
Age	< 65	569	31 (38.3)	538 (41.4)	0.5735
	≥ 65	810	50 (61.7)	760 (58.6)	
Sex	Male	573	44 (54.3)	529 (40.8)	**0.0162**
	Female	806	37 (45.7)	769 (59.2)	
Stage	I	847	33 (40.7)	814 (62.7)	**0.0022**
	II	264	26 (32.1)	238 (18.3)	
	III	222	19 (23.5)	203 (15.6)	
	IV	46	3 (3.7)	43 (3.3)	
Tumor mutational burden	Low	1060	42 (51.9)	1018 (78.4)	**< 0.0001**
	High	319	39 (48.1)	280 (21.6)	
Smoking history	Yes	1055	74 (91.4)	981 (75.6)	**0.0011**
	No	324	7 (8.6)	317 (24.4)	

Bold indicates highlight statistically significant results.

**Table 2:** Univariate and multivariate analysis of lung adenocarcinoma.

Characteristics	Total (*N*)	Univariate analysis		Multivariate analysis	
		Hazard ratio (95% CI)	*P*‐value	Hazard ratio (95% CI)	*P*‐value
*SMARCA4*	1379				
Wild‐type	1298	Reference		Reference	
Mutant	81	2.169 (1.495–3.147)	**< 0.001**	1.539 (1.045–2.269)	**0.029**
Sex	1379				
Female	806	Reference		Reference	
Male	573	1.550 (1.238–1.941)	**< 0.001**	1.163 (0.921–1.468)	0.203
Age	1379				
< 65	569	Reference		Reference	
≥ 65	810	1.297 (1.030–1.635)	**0.027**	1.449 (1.147–1.832)	**0.002**
Stage	1379				
I	847	Reference		Reference	
II	264	2.262 (1.685–3.035)	**< 0.001**	2.275 (1.688–3.065)	**< 0.001**
III	222	3.290 (2.490–4.347)	**< 0.001**	3.489 (2.627–4.635)	**< 0.001**
IV	46	7.437 (4.962–11.148)	**< 0.001**	7.873 (5.204–11.911)	**< 0.001**
Tumor mutational burden	1379				
High	319	Reference		Reference	
Low	1060	0.770 (0.595–0.997)	**0.047**	1.067 (0.808–1.408)	0.649
Smoking history	1379				
Yes	1055	Reference		Reference	
No	324	0.544 (0.409–0.724)	**< 0.001**	0.526 (0.387–0.714)	**< 0.001**

Bold indicates highlight statistically significant results.

### *SMARCA4* is involved in a variety of biological processes in LUAD

**Fig. 4:**
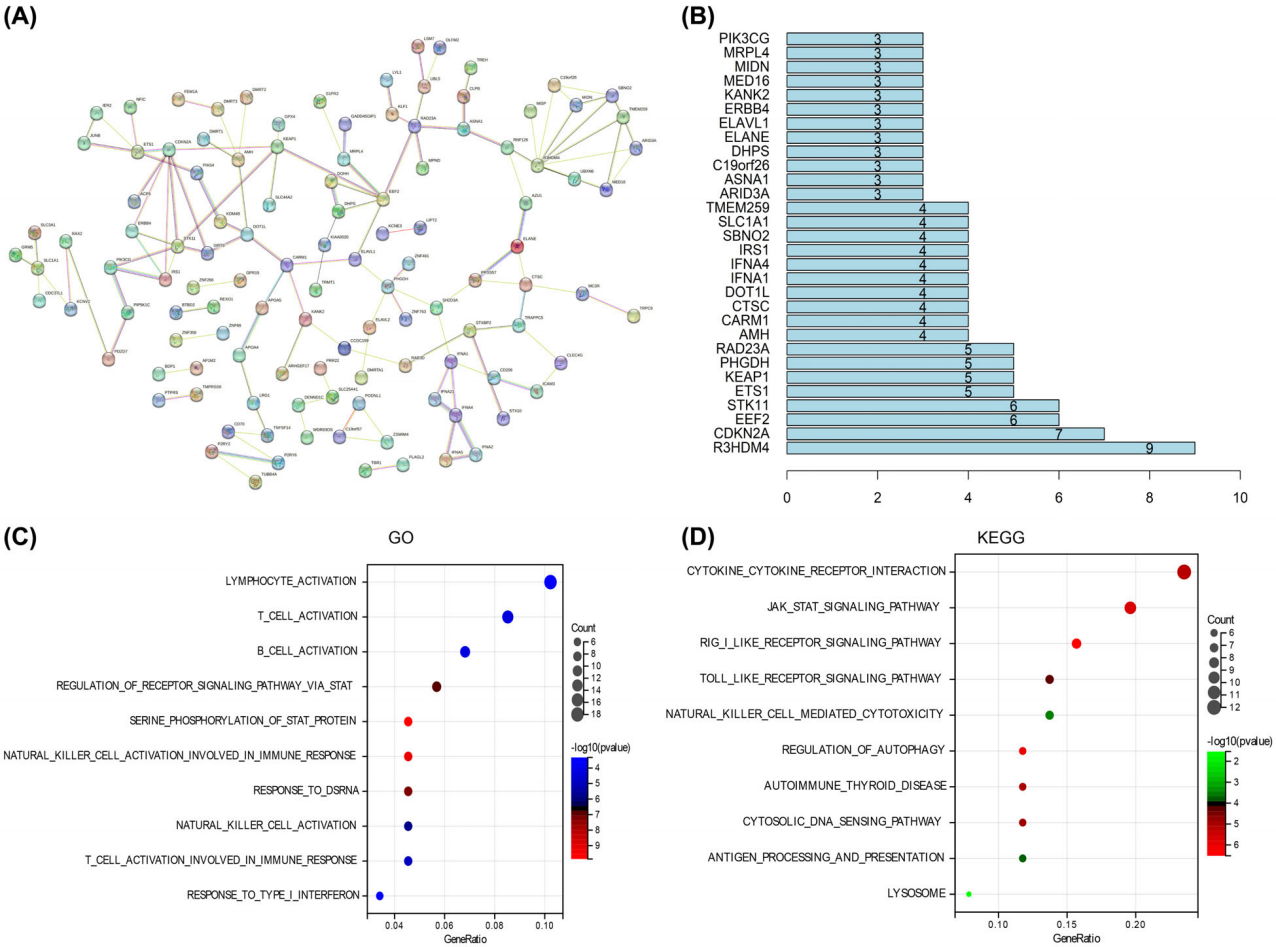
Gene set enrichment analysis of lung adenocarcinoma (LUAD). (A) Protein–protein interaction (PPI) network based on *SMARCA4* mutations in LUAD. (B) Core proteins in the PPI network. (C, D) Gene Ontology (GO) and Kyoto Encyclopedia of Genes and Genomes (KEGG) enrichment analyses of *SMARCA4* mutations in LUAD.

To investigate the mechanism by which *SMARCA4* mutations affect LUAD, we screened the top 200 genes that were most strongly correlated with *SMARCA4* in the *SMARCA4*‐altered group. Using the STRING tool, we constructed a PPI network (Fig. 4A) and identified two central proteins (R3HDM4 and CDKN2A) with the highest node degrees (Fig. 4B). Gene Ontology (GO) (http://geneontology.org) and Kyoto Encyclopedia of Genes and Genomes (KEGG) (https://www.genome.jp/kegg) enrichment analyses were performed to identify the potential pathways influenced by *SMARCA4* mutations. The GO enrichment analysis results showed that *SMARCA4* might be involved in pathways such as ‘lymphocyte activation’, ‘regulation of receptor signaling pathway via STAT’, ‘T‐cell activation involved in immune response’ and ‘response to type I interferon’ (Fig. 4C). The KEGG enrichment results indicated that *SMARCA4* was associated with pathways such as ‘cytokine–cytokine receptor interaction’, ‘Jak‐STAT signaling pathway’, ‘Toll‐like receptor signaling pathway’, ‘natural killer cell‐mediated cytotoxicity’ and ‘regulation of autophagy’ (Fig. 4D).

### The efficacy of immunotherapy in *SMARCA4*‐mutant LUAD is associated with the *KRAS*, *STK11* and *TP53* mutation status

**Fig. 5:**
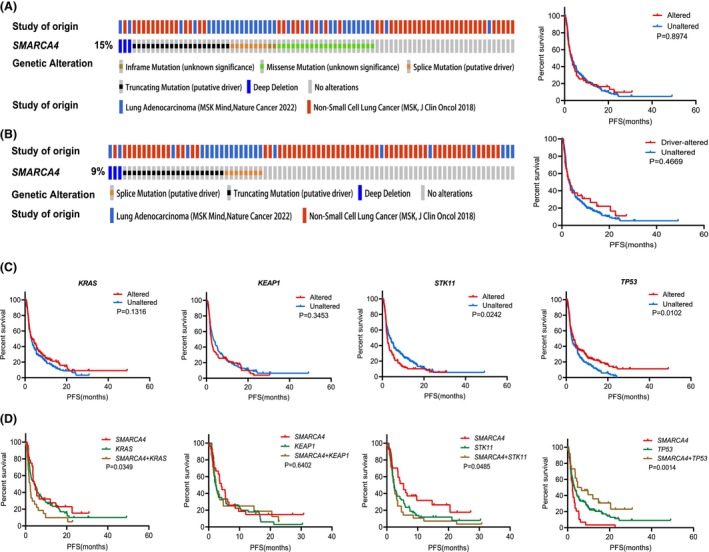
*SMARCA4* mutation status and prognosis based on two lung adenocarcinoma (LUAD) immunotherapy datasets. (A) Distribution of *SMARCA4* mutations in LUAD immunotherapy datasets and progression‐free survival (PFS) after immunotherapy in the *SMARCA4*‐altered and *SMARCA4*‐unaltered groups. (B) Distribution of *SMARCA4* driver mutations in LUAD immunotherapy datasets and the PFS after immunotherapy in the *SMARCA4* driver‐altered and *SMARCA4*‐unaltered groups. (C) Survival curves of immune checkpoint inhibitors (ICIs)‐treated patients stratified by the mutational status of *KRAS*, *KEAP1*, *STK11* and *TP53*. (D) Survival curves of ICIs‐treated patients stratified by the combination of *SMARCA4* mutations and mutations in *KRAS*, *KEAP1*, *STK11* and *TP53*.

Recently, multiple studies have confirmed the correlation between *SMARCA4* alterations and immunotherapeutic effectiveness in lung cancer, but the conclusions remain controversial. Table 3 lists the available relevant studies. Therefore, we explored the effect of *SMARCA4* mutations on the immune response in patients with LUAD. We selected 366 LUAD samples from two lung cancer immunotherapy datasets in cBioPortal; 15% (55/366) of the patients had *SMARCA4* mutations (Fig. 5A), including 32 patients (9%) with driver mutations (Fig. 5B). K–M survival analysis showed no significant difference in PFS between the *SMARCA4*‐altered (mPFS: 3.5 months) and *SMARCA4*‐unaltered (mPFS: 3.03 months) groups (*P* = 0.8974) (Fig. 5A). In addition, no significant difference in PFS was observed between the *SMARCA4*‐driver‐altered (mPFS: 3.35 months) and the unaltered (mPFS: 3.07 months) groups (*P* = 0.4183) (Fig. 5B).

**Table 3:** Impact of SMARCA4 on ICIs treatment outcomes for patients with lung cancer. ICIs, immune checkpoint inhibitors; LUAD, lung adenocarcinoma; NA, not available; NSCLC, non‐small cell lung cancer; ORR, objective response rate; OS, overall survival; PFS, progression‐free survival; PR, partial response; SCLC, small‐cell lung cancer; SMARCA4‐d, SMARCA4‐deficient; *SMARCA4*‐m, *SMARCA4*‐mutant.

Disease	Report type	Case number				Outcome				References
		Total	*SMARCA4*‐m	SMARCA4‐d (*n*/total)	ICIs	OS	PFS	ORR	Prognosis	
NSCLC	Retrospective	28 703	3305	29/64	11	NA	35 days	NA	Clinical outcomes were poor in SMARCA4‐d NSCLC	[47]
NSCLC	Retrospective	4813	407	50/86	Overall response was assessed in 445 of 570 patients	No difference	No difference	Class I alterations had a higher ORR	No difference in PFS and OS	[45]
NSCLC	Retrospective	326	15/19^a^	105/326	43	Not reached	NA	NA	Patients treated with ICIs plus chemotherapy had a longer mOS	[22]
SCLC	Case report	1	NA	1/1	1	Not applicable	Not applicable	Not applicable	Hyperprogressive	[61]
NSCLC	Retrospective	29 135	3381	NA	NA	Homozygous truncated: 9.9 months	NA	NA	Patients with NSCLC with homozygous *SMARCA4* alterations had worse outcomes	[49]
KRAS‐mutant LUAD	Retrospective	564	54	NA	95	NA	1.73 months	NA	Poor survival in *KRAS*–*SMARCA4*‐co‐mutant cases	[28]
Thoracic sarcoma	Case report	3	3	3/3	3	Not applicable	Not applicable	Not applicable	ICIs had a good effect	[62]
NSCLC	Retrospective	2689	164/1490^b^	NA	57/532	11.0 months	2.1 months	22.8%	No associations between *SMARCA4* mutations and ICIs efficacy, but poor survival in *KRAS*–*SMARCA4*‐co‐mutant cases	[52]
Thoracic sarcoma	Case report	1	1	1/1	1	Not Applicable	Not Applicable	Not Applicable	Dramatic regression	[63]
NSCLC	Retrospective	534	42/63^c^	63/534	7	< 18 months	NA	NA	Patients with SMARCA4‐d NSCLC had a shorter OS than those with non‐SMARCA4‐d NSCLC	[64]
Thoracic undifferentiated tumor	Case report	1	1	1/1	1	Not applicable	Not applicable	Not applicable	PR after 20 days and after six cycles of tislelizumab demonstrated a sustained durable PR response	[65]
Thoracic tumors	Retrospective	45	NA	45/45	8	NA	NA	50%	ICIs can be considered an effective treatment option for SMARCA4‐d tumors	[66]

^a^ *SMARCA4* gene mutations were present in 15 of 19 SMARCA4‐d NSCLC cases with available next‐generation sequencing data

^b^ Among the 1490 cases with advanced NSCLC, 11.0% (*N* = 164) had *SMARCA4* mutations

^c^ Forty‐two of the 63 patients with SMARCA4‐d NSCLC had at least one *SMARCA4* mutation.

Given that *SMARCA4* mutations often co‐occur with mutations in *KRAS*, *KEAP1*, *STK11* and *TP53*, we analyzed the effects of these four gene mutations on PFS following immunotherapy. The results showed that patients with *STK11* mutations exhibited poorer responses to immunotherapy than those in the unaltered group (mPFS: 2.3 months *vs*. 3.6 months, *P* = 0.0242), whereas patients with *TP53* mutations were more sensitive to immunotherapy (mPFS: 3.5 months *vs*. 2.6 months, *P* = 0.0102). Additionally, *KRAS* or *KEAP1* mutations were not significantly associated with the immunotherapy efficacy (Fig. 5C). Next, we analyzed the efficacy of immunotherapy in the context of *SMARCA4* and *KRAS*, *KEAP1*, *STK11* or *TP53* co‐occurring mutations (Fig. 5D and Fig. S2A). Among patients treated with immunotherapy, the mPFS of those with both *SMARCA4* and *KRAS* mutations was significantly shorter than that of patients with mutations in either gene alone (*SMARCA4* + *KRAS*: 2.1 months, *SMARCA4*: 4.4 months, *KRAS*: 4.33 months; *P* = 0.0349). Compared with that of patients with *SMARCA4* mutations alone (mPFS: 5.4 months), the mPFS of patients with *SMARCA4* and *STK11* mutations was significantly shorter (mPFS: 2.3 months). By contrast, when *SMARCA4* and *TP53* mutations were both present, the response to immunotherapy was superior to that in patients with mutations in either gene alone (*SMARCA4* + *TP53*: 5.65 months, *SMARCA4*: 2.3 months, *TP53*: 3.17 months; *P* = 0.0014). However, the presence or absence of *SMARCA4* mutations did not affect the immunotherapy response of patients with LUAD when *KEAP1* mutations were present (*P* = 0.6402). We further analyzed the effectiveness of different gene mutations within the *SMARCA4* mutation subgroup on the prognosis of patients who received immunotherapy. Consistent with the aforementioned results, in the *SMARCA4*‐altered subgroup, patients with *KRAS* or *STK11* mutations exhibited a significantly shorter PFS than those without these mutations, whereas patients with *TP53* mutations had a significantly longer PFS. However, the presence or absence of *KEAP1* mutations did not affect the PFS of patients with LUAD after immunotherapy (Fig. S2B). Because PD‐L1 is an important therapeutic target in immunotherapy, we analyzed its expression in different groups. The results showed that PD‐L1 expression was significantly decreased in the *STK11*‐altered group and significantly increased in the *TP53*‐altered group, whereas it was not associated with *KRAS* or *KEAP1* mutations (Fig. S2C). No significant difference was observed in PD‐L1 expression between the *SMARCA4*‐altered group and the unaltered group (Fig. S2D). Additionally, within the *SMARCA4*‐altered subgroup, PD‐L1 expression was not significantly associated with mutations in *KRAS*, *KEAP1*, *STK11* or *TP53* (Fig. S2E).

In the POPLAR and OAK studies, the presence or absence of *SMARCA4* mutations did not affect the overall prognosis of patients with NSCLC. Furthermore, no statistically significant difference was noted in the mOS or mPFS between the two groups (*P* > 0.05) (Fig. S3A,B). In patients who received immunotherapy, there was no statistically significant difference in OS or PFS between the altered and unaltered groups (Fig. S3C,D). Moreover, within the altered group, no significant difference was observed in survival between patients who received chemotherapy and those who received immunotherapy (Fig. S3E,F). Among the patients with *SMARCA4* mutations, 35 had driver mutations, and of these patients, 15 received atezolizumab immunotherapy and 20 received docetaxel chemotherapy. However, survival analysis showed no statistically significant differences between patients with driver mutations and those with non‐driver mutations or between patients treated with immunotherapy and those treated with chemotherapy (Fig. S3G–L). Thus, the population and sample sizes of the studies might have led to different conclusions.

### Low *SMARCA4* expression is associated with poor LUAD prognosis but might indicate a benefit with immunotherapy

**Fig. 6:**
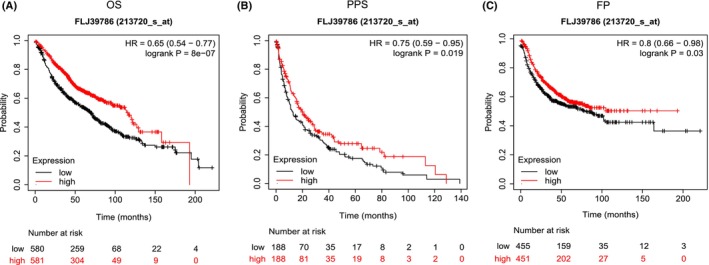
Relationships between *SMARCA4* expression and the prognosis of patients with lung adenocarcinoma (LUAD) according to the Kaplan–Meier (K–M) plotter. (A) Overall survival (OS). (B) Post progression survival (PPS). (C) First progression (FP).

**Fig. 7:**
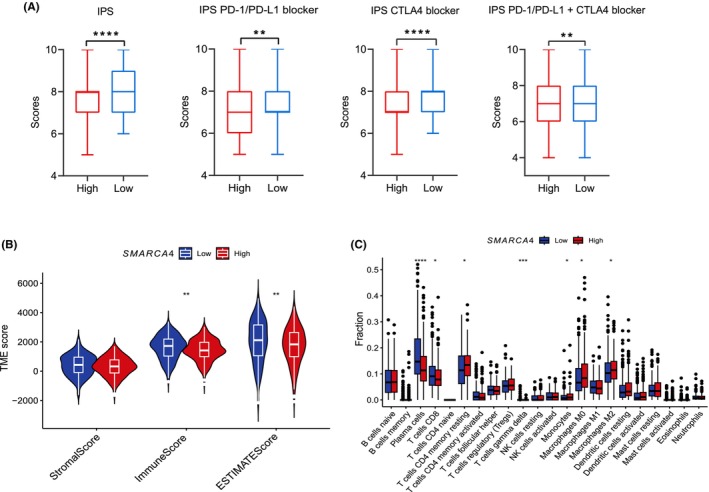
Relationship between *SMARCA4* expression and the tumor microenvironment (TME) of lung adenocarcinoma (LUAD). (A) Association between *SMARCA4* expression and immunotherapy response in patients with LUAD based on The Cancer Genome Atlas (TCGA) database (minimum to maximum, Student's *t*‐test, high: *n* = 263, low: *n* = 252). (B) Relationships between *SMARCA4* expression and the TME score of patients with LUAD based on TCGA database (minimum to maximum, Wilcoxon rank‐sum test, high: *n* = 269, low: *n* = 270). (C) Correlation between *SMARCA4* expression and immune cell infiltration levels in LUAD (minimum to maximum, wilcoxon rank‐sum test, high: *n* = 269, low: *n* = 270). *P < 0.05; **P < 0.01; ***P < 0.001; ****P < 0.0001.

Because *SMARCA4* mutations lead to gene expression downregulation, we analyzed the relationship between *SMARCA4* expression and the prognosis of patients with LUAD. Using the K–M plotter online tool, we found that the high‐*SMARCA4* expression group had a significantly better prognosis than the low‐*SMARCA4* expression group (Fig. 6). We then obtained the gene expression profiles of patients with LUAD from TCGA database and divided the patients into high‐ and low‐expression groups according to the median *SMARCA4* mRNA level. We also downloaded the TCGA‐LUAD immune score file from the TCIA. As shown in Fig. 7A, regardless of the application of PD‐1/PD‐L1 inhibitors or cytotoxic T‐lymphocyte‐associated antigen 4 inhibitors, the *SMARCA4* low‐expression group had a higher immunophenoscore, which indicates that LUAD patients with low *SMARCA4* expression might benefit from immunotherapy. Furthermore, we analyzed the TME of the two groups and found that both the immune and ESTIMATE scores were greater in the low‐expression group (Fig. 7B). Additionally, compared with that in the high‐expression group, the infiltration of plasma cells, CD8+ T cells, and gamma delta T cells was significantly greater in the low‐expression group, whereas the infiltration of Treg cells, M0 macrophages and M2 macrophages was lower (Fig. 7C).

## Discussion

As a tumor suppressor, *SMARCA4* mutations and low expression are associated with a poor prognosis in various cancers [47, 48]. In the present study, 14 datasets containing data on patients with LUAD were used to systematically analyze the clinical characteristics and prognostic impact of *SMARCA4* mutations, with a particular emphasis on the potential of *SMARCA4* to serve as a prognostic marker for LUAD. Compared with other SWI/SNF family members, *SMARCA4* exhibited a greater mutation frequency, and its mutations were associated with a poorer prognosis, which is consistent with previous research [22, 45]. Furthermore, *SMARCA4* mutations and *ARID1A* mutations were mutually exclusive, which suggests a better genetic background for further research on *SMARCA4*.

In the present study, *SMARCA4* mutations resulted in decreased gene expression, which suggests that such mutations might lead to gene inactivation. Missense and truncating mutations accounted for most of the mutations; truncating mutations were the main driver mutations, but splice and missense mutations also played a role. Although multiple studies have confirmed that among various *SMARCA4* mutation types, truncating mutations are more likely to result in gene function inactivation and that the functional consequences of missense mutations are not fully understood, Fernando *et al*. [49] reported that some missense mutations significantly suppress remodeling activity of the SWI/SNF complex. Loss of the SMARCA4 protein is not exclusive to tumors with frameshift or nonsense *SMARCA4* mutations because some missense mutations have been shown to be deleterious [46, 50, 51]. Therefore, we cannot rule out the oncogenic role played by missense mutations. We also conducted a prognostic analysis according to the two mutation types. The results suggested no significant difference in prognosis between patients with class I mutations and those with class II mutations, but patients with LUAD with either mutation type had a poorer prognosis than those with wild‐type *SMARCA4*. This could be because some missense class II mutations also function as driver mutations and result in gene dysfunction [49, 50, 51].

In the present study, *SMARCA4* mutations were more prevalent among male patients and were closely associated with smoking. *SMARCA4* mutations were also correlated with a later clinical stage, higher TMB and higher MSI score, which is consistent with previous research [45, 52]. The presence of *SMARCA4* mutations, which is an independent risk factor for LUAD, indicates a poor clinical prognosis and provides a potential target for clinical diagnoses and treatment. Additionally, our study showed that the smoking status, age, and stage were also independent prognostic factors for LUAD. As expected, those patients who were older, had a later disease stage and had a history of smoking demonstrated a worse prognosis.

A series of studies have indicated that SWI/SNF mutations, particularly *SMARCA4* mutations, are often accompanied by mutations in *TP53*, *KRAS*, *KEAP1*, *STK11* and other genes in lung cancer. However, they are mutually exclusive with the most common driver gene mutations, such as those in *EGFR*, *ALK* and *MET*, which was confirmed in our research [45, 49, 53]. In addition, our study suggested that mutations in *KEAP1*, *STK11* and *CDKN2A* are most strongly correlated with *SMARCA4* mutations and that CDKN2A is central in the PPI network.

Currently, PD‐L1 expression levels and TMB are widely‐used biomarkers to determine immunotherapy eligibility. Many patients have benefited from this approach, but some patients still exhibit immune resistance. Several studies have reported a close association between SWI/SNF mutations in tumors and sensitivity to immunotherapy [54], but the relationship between *SMARCA4* mutations or expression and immunotherapy efficacy remains controversial. Studies have shown that SMARCA4 deficiency increases the infiltration of anti‐tumor immune cells, thereby improving the response to immunotherapy [55]. Naito T *et al*. reported a case of a patient with SMARCA4‐deficient LUAD, negative for driver gene mutations, with high PD‐L1 expression and a high TMB. This patient showed a sustained response to fourth‐line nivolumab for 14 months [56]. Furthermore, some studies have indicated that *SMARCA4* mutations do not increase the response to immunotherapy. However, in the presence of *SMARCA4* and *KRAS* mutations, a shorter survival period was previously observed, which could be attributed to the lower PD‐1 expression and infiltration of CD8+ T cells in the tumor stroma [52]. In our study, patients in the *SMARCA4*‐altered group did not benefit more from immunotherapy. However, when a *SMARCA4* mutation was accompanied by a *KRAS* or *STK11* mutation, immunotherapy resistance occurred. A further immunogenomic analysis suggested that patients with mutations in *SMARCA4* and genes such as *KEAP1* or *STK11* exhibit an immune‐cold phenotype, which results in resistance or a lack of response to immunotherapy [57, 58]. By contrast, for co‐existing *SMARCA4* and *TP53* mutations, patients with LUAD had a longer PFS. Studies have shown that *TP53* mutations are associated with increased PD‐L1 expression and an increased TMB. In addition, *TP53* mutations promote CD8+ T cell infiltration, which could be a reason for increased sensitivity to immunotherapy [59, 60]. In the present study, we investigated the correlation between PD‐L1 and *SMARCA4* mutations, as well as other genomic mutations. However, our results did not show significant differences, which might be a result of the small sample size of the immunotherapy dataset.

In addition to *SMARCA4* mutations, we used the immunophenoscore to predict the correlation between *SMARCA4* expression and the response to immunotherapy based on the TCGA‐LUAD dataset. Patients in the low‐*SMARCA4* expression group might exhibit a better response to immunotherapy. The immune response can also be explained via an analysis of the TME. The low expression of *SMARCA4* might enhance the anti‐tumor immune response by regulating the TME, which could increase the infiltration of anti‐tumor immune cells, such as CD4+ T cells and CD8+ T cells. However, because of the lack of immunotherapy survival data, we were unable to reach more direct prognostic conclusions, which is a limitation of our study. Through GO and KEGG enrichment analyses, we found that *SMARCA4* mutations might activate lymphocyte‐ and immune‐related pathways. These results also suggest that *SMARCA4* mutations or low expression can positively regulate the TME. Although most *SMARCA4* mutations result in gene inactivation and the downregulation of its expression, some mutations do not decrease gene expression [45], which might also affect the efficacy of ICIs in patients with *SMARCA4* mutations.

Most previous studies in this field have focused on SMARCA4‐deficient thoracic tumors or NSCLC, whereas our study explored the potential value of *SMARCA4* as a prognostic marker in LUAD, which has clear innovative and clinical application value. Notably, our research also has some limitations. First, although we analyzed *SMARCA4* mutations and mRNA level data from multiple datasets, the correlations with protein levels require further validation. Second, the small sample size and limited data in the immunotherapy dataset restricted the exploration of immune response‐related characteristics, which might have also contributed to the differences in the results. In addition, a multivariate analysis of factors that affect the immunotherapy response was not performed using valid clinical data, which was not statistically rigorous.

## Conclusions

In summary, by combining multiple datasets, our data suggest that *SMARCA4* mutations and low expression are associated with poor prognosis in patients with LUAD and could serve as critical prognostic biomarkers. Although low *SMARCA4* expression might enhance the response to immunotherapy by improving the anti‐tumor immune microenvironment, further studies with larger samples are needed to confirm these findings. Moreover, attention should be given to genes co‐mutant with *SMARCA4* because patients with *KRAS* or *STK11* mutations exhibit poorer responses to immunotherapy, whereas those with *TP53* mutations show better responses. These findings might provide new insights for clinical LUAD diagnosis and treatment.

## Conflicts of interest

The authors declare that they have no conflicts of interest.

### Peer review

The peer review history for this article is available at https://www.webofscience.com/api/gateway/wos/peer‐review/10.1002/2211‐5463.13899.

## Author contributions

HH contributed to conception/design. HH and YZ contributed to provision of study material or patients. YZ, DS, WH, ZY and CZ contributed to collection and/or assembly of data. YZ, DS, YL, XZ, YW, CZ and NL contributed to data analysis and interpretation. HH and YZ contributed to manuscript writing. All authors approved the final version of the manuscript submitted for publication.

## Supporting information


**Fig. S1.** Relationships between SWI/SNF subunit mutations and the prognosis of patients with lung adenocarcinoma.
**Fig. S2.** Immunotherapy survival curves and PD‐L1 expression among subgroups.
**Fig. S3.** Prognostic analysis of NSCLC patients with *SMARCA4* mutations in the POPLAR study and the OAK study.

## Data Availability

All the data generated during this study are included in this published article. The datasets used in the current study are available from the cBioPortal for Cancer Genomics (https://www.cbioportal.org) and The Cancer Genome Atlas (TCGA) (https://portal.gdc.cancer.gov).
