# Supplementary material for: SMARCA4 mutations and expression in lung adenocarcinoma: prognostic significance and impact on the immunotherapy response
Source: FEBS Open Bio. 2024 Sep 25;14(12):2086–103. doi: 10.1002/2211-5463.13899 (PMC11609588; doi:10.1002/2211-5463.13899)
Supplement: Supplementary file 1 — Fig. S1. Relationships between SWI/SNF subunit mutations and the prognosis of patients with lung adenocarcinoma. Fig. S2. Immunotherapy survival curves and PD‐L1 expression among subgroups. Fig. S3. Prognostic analysis of NSCLC patients with SMARCA4 mutations in the POPLAR study and the OAK study. [file FEB4-14-2086-s001.pdf]

Supplemental information

SMARCA4 mutations and expression in lung adenocarcinoma: prognostic significance and impact on the immunotherapy response

Supplementary Figure 1. Relationships between SWI/SNF subunit mutations and the prognosis of patients with lung adenocarcinoma.

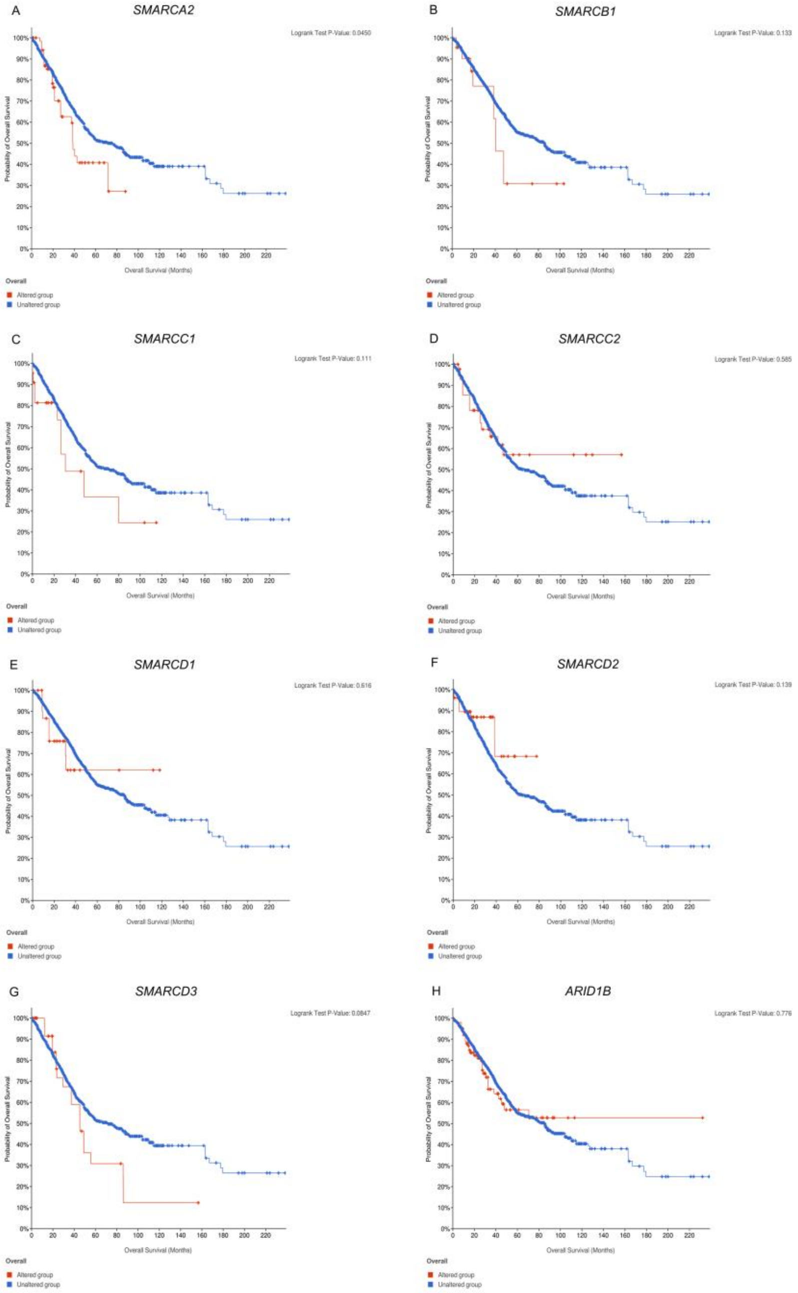

Relationships between SWI/SNF subunit mutations and the prognosis of patients with lung adenocarcinoma. A. *SMARCA2*; B. *SMARCB1*; C. *SMARCC1*; D. *SMARCC2*; E. *SMARCD1*; F. *SMARCD2*; G. *SMARCD3*; H. *ARID1B*.

Supplementary Figure 2. Immunotherapy survival curves and PD-L1 expression among subgroups.

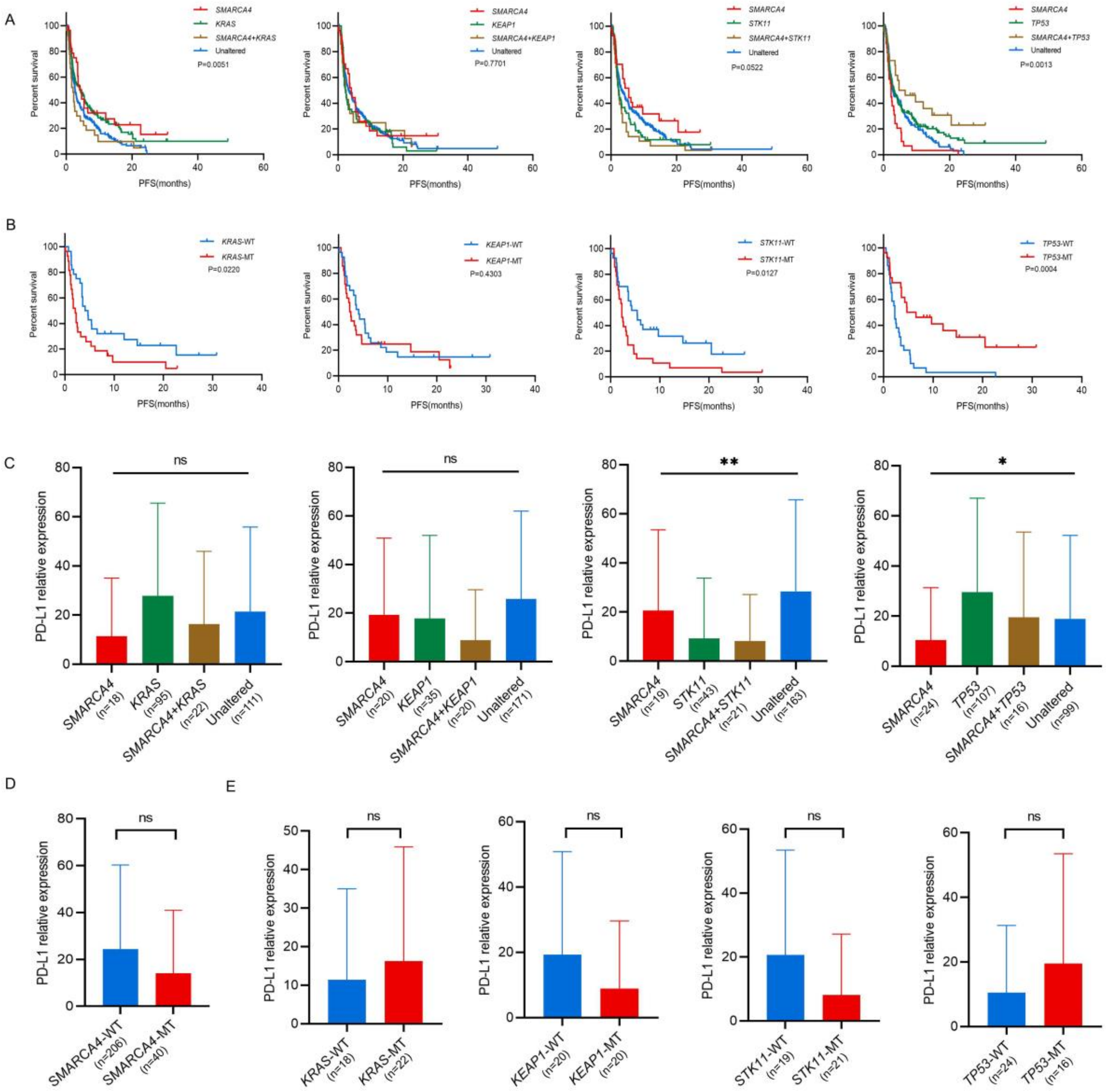

A. Survival curves of immune checkpoint inhibitors treated patients based on the combination of *SMARCA4* mutations and *KRAS*, *KEAP1*, *STK11*, and *TP53* mutations. B. The relationships between *KRAS*, *KEAP1*, *STK11*, and *TP53* mutations and PFS in the *SMARCA4*-altered group. C. PD-L1 expression in different subgroups (mean ± SD, one-way ANOVA). D. Relationships between *SMARCA4* mutations and PD-L1 expression in patients with lung adenocarcinoma (mean ± SD, student's *t*-test). E: Relationships between *KRAS*, *KEAP1*, *STK11*, and *TP53* mutations and PD-L1 expression in the *SMARCA4*-altered group (mean ± SD, student's *t*-test). WT: wild type; MT: mutant type; ns: P > 0.05; *: P<0.05; **: P<0.01.

Supplementary Figure 3. Prognostic analysis of NSCLC patients with *SMARCA4* mutations in the POPLAR study and the OAK study.

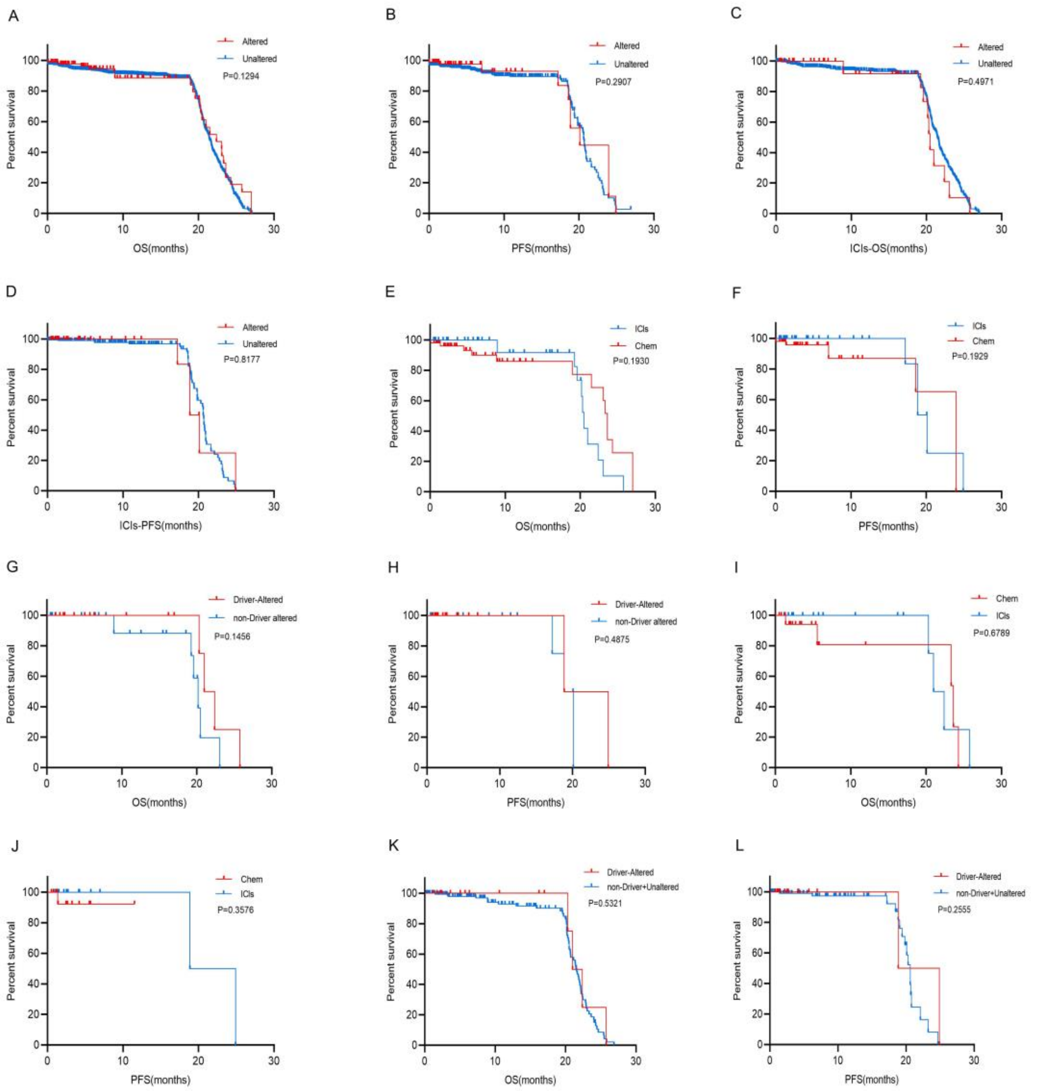

A-B. OS and PFS of the *SMARCA4*-altered group and the *SMARCA4*-unaltered group; C-D. Survival analysis of patients in the *SMARCA4*-altered group and the *SMARCA4*-unaltered group who received immunotherapy; E-F. OS and PFS of patients in the *SMARCA4*-altered group who received chemotherapy and immunotherapy; G-H. OS and PFS of patients in the *SMARCA4*-driver-altered group and *SMARCA4*-non-driver-altered group who received immunotherapy; I-J. OS and PFS of patients in the *SMARCA4* driver-altered group who received chemotherapy and immunotherapy; K-L. OS and PFS of patients in the *SMARCA4*-driver-altered group and *SMARCA4*-non-driver-altered and unaltered group who received immunotherapy.
